# Stroke-CORE: master study protocol of the national cohort of the specialty network stroke within the network university medicine in Germany

**DOI:** 10.1186/s42466-026-00547-8

**Published:** 2026-10-30

**Authors:** Flora Noelle Wiegand, Angelika Alonso, Heinrich J. Audebert, Jessica Barlinn, Martin Bendszus, Folke Brinkmann, Bastian Cheng, Mark Dzietko, Katharina Feil, Agnes Flöel, Christina Franzisket, Lucia Gerstl, Christian Grefkes, Kai Günther, Kathrin Harringa, Steffi Jiru-Hillmann, Johanna-Luise Kanwisch, Lucia Kerti, Martin Köhrmann, Anna Kopczak, Claudia Kunze, Jennifer Linn, Annerose Mengel, Dominik Michalski, Jens Minnerup, Christian H. Nolte, Christian Otte, Jan Purrucker, Dorothee Saur, Wolf Schäbitz, Stefan Schwab, Kristina Szabo, Timo Uphaus, Rolf Wachter, Markus Wagner, Peter U. Heuschmann, Karen Gertz, Götz Thomalla

**Affiliations:** 1 University Hospital Würzburg, Würzburg, Germany; 2 Universitätsmedizin Mannheim, Heidelberg University, Mannheim, Germany; 3 Charite Universitatsmedizin Berlin, Berlin, Germany; 4 Medizinische Fakultät und Universitätsklinikum Carl Gustav Carus, Technische Universität Dresden, Dresden, Germany; 5 Abteilung Für Neuroradiologie, Universitätsklinikum Heidelberg, Heidelberg, Germany; 6 Universitätsklinikum Schleswig-Holstein, Kiel, Germany; 7 Universitätsklinikum Hamburg-Eppendorf (UKE), Hamburg, Germany; 8 Universitätsklinikum Düsseldorf, Düsseldorf, Germany; 9 Universitätsklinikum Ulm, Ulm, Germany; 10 Department of Neurology, University Medicine Greifswald, Greifswald, Germany; 11 German Center for Neurodegenerative Diseases, Site Rostock/Greifswald, Greifswald, Germany; 12 Stiftung Deutsche Schlaganfall-Hilfe, Gütersloh, Germany; 13 Division of Pediatric Neurology and Developmental Medicine, Department of Pediatrics, Hauner Children’s Hospital, Ludwig-Maximilians-University (LMU) Hospital, LMU Munich, Dr. Von, Munich, Germany; 14 Center for Children With Medical and Developmental Complexity, Munich University, LMU Hospital, LMU Munich, Munich, Germany; 15 Universitätsmedizin Frankfurt, Frankfurt, Germany; 16 Department of Neurology and Center for Translational Neuro- and Behavioral Sciences (C-TNBS), University Hospital Essen, Essen, Germany; 17 Institute for Stroke and Dementia Research (ISD), LMU University Hospital, LMU Medizin, LMU Munich, Munich, Germany; 18 Institut Und Poliklinik Für Diagnostische Und Interventionelle Neuroradiologie, Medizinische Fakultät und Universitätsklinikum Carl Gustav Carus, Technische Universität Dresden, Dresden, Germany; 19 Universitätsklinikum Tübingen, Tübingen, Tübingen, Germany; 20 Universitätsklinikum Leipzig, Leipzig, Germany; 21 Department of Neurology, University of Luebeck, Luebeck, Germany; 22 DKFZ (German Centre for Cardiovascular Research), Partner Site North, Luebeck, Germany; 23 Universitätsklinikum Heidelberg, Heidelberg, Germany; 24 Evangelisches Klinikum Bethel, Universitätsklinik Für Neurologie, Universität Bielefeld, Bielefeld, Germany; 25 Universitätsklinikum Erlangen, Erlangen, Germany; 26 Department of Neurology, Johannes Gutenberg University, Mainz, Germany; 27 Department of Cardiology, University Hospital Leipzig, Leipzig, Germany

**Keywords:** Stroke, Research infrastructure, Real-world data, Ischaemic stroke, Intracerebral haemorrhage, Paediatric stroke

## Abstract

**Introduction:**

Although Germany has a strong history of stroke research, it currently lacks a national harmonised infrastructure for clinical stroke research. To address this gap, the stroke-specific Specialty Network (SN Stroke) of the Network University Medicine (NUM) was established as a comprehensive research platform. Hereby we present the protocol of the Stroke-CORE cohort (COhort for REsearch in Stroke) within SN Stroke enrolling a large representative cohort of patients with acute ischeamic stroke or intracerebral haemorrhage in Germany.

**Methods:**

Stroke-CORE is a national, prospective, longitudinal, multi-centre cohort study with a decentralised biobank, structured as a modular umbrella protocol. Adult patients with acute ischaemic stroke or intracerebral haemorrhage, and paediatric stroke patients are enrolled at participating hospitals. Data collection follows four adaptive levels, ranging from a core clinical data set (Level 1) to modules covering the whole stroke care pathway (Level 2), biosamples (Level 3), and voluntary additional modules (Level 4), with follow-up at discharge, 90 days, 180 days (optional), and annually. Enrolment started in August 2026 across 12 initial sites, with a target of 12,750 patients being recruited by 2030.

**Perspective:**

Stroke-CORE represents a nationally harmonised cohort spanning prehospital care, acute treatment, rehabilitation, prevention, aftercare, and paediatric stroke. Its adaptive modular design allows continuous expansion with new modules, data streams, and biobanking initiatives without protocol revision. As a sustainable national research platform, Stroke-CORE aims to address key knowledge gaps, facilitate recruitment for clinical trials, and accelerate translational, clinical and outcomes research.

**Trial registration:**

The protocol is registered at German Clinical Trials Register (DRKS00039775, July 8th, 2026).

## Background

In Germany, approximately 250,000 new strokes occur annually [1], and about 1.8 million people are living with the consequences of a stroke [2].

Beyond its substantial clinical burden, stroke poses a major economic burden on the German healthcare system and society, with first-year costs ranging from approximately €15,137 and €22,666 for cerebral infarction [3–5] and €23,886 for intracerebral haemorrhage [5], to €43,184 and €44,057 for SAH per patient depending on stroke subtype and severity [6, 7] health care costs per inhabitant for cerebral infarction (ICD-10 I63) was estimated to be €100 in 2023 [8]. Given an aging population, these costs are expected to rise further, underlining the need for harmonised research infrastructures to accelerate the generation of evidence for more effective prevention, treatment, and long-term care.

Although substantial advances in stroke care have been achieved in recent years especially in acute ischaemic stroke including endovascular thrombectomy [9], evidence-based treatment options remain limited across various stages of the stroke care continuum, particularly for specific subtypes such as intracerebral haemorrhage and post-stroke complications such as cognitive impairment and depression. Furthermore, interventions of trials targeting post-stroke care and secondary prevention have not yet translated into improvements in patient outcomes or reducing stroke recurrence rates. These challenges are expected to increase in relevance given the growing incidence and prevalence of stroke associated with older age [10].

Paediatric stroke, although uncommon, is associated with severe long-term neurological impairment [11]. Limited knowledge of its aetiologies and disease mechanisms continues to hamper prevention, diagnosis, treatment, and long-term care [12].

Germany provides highly specialised stroke care through a dense network of qualified acute stroke units and research-active centres with a strong track record of national and international trials [13–17]. However, in contrast to many other countries with established national stroke research infrastructures such as the USA [18], France [19], or the United Kingdom [20, 21], Germany still lacks a national structured and harmonised infrastructure to support large-scale clinical stroke research. To address this gap, the Specialty Network Stroke (SN Stroke) within the Network University Medicine (NUM) was recently founded. Within SN Stroke, the Stroke-CORE (COhort for REsearch in Stroke) will be established as a nationwide, prospective, longitudinal cohort study to expedite medical research breakthroughs across the entire stroke care pathway, from prehospital stroke management to post stroke aftercare, and across all age groups.

Building on existing German stroke registries, which primarily focus on quality assurance in acute stroke care, Stroke-CORE is designed as a nationwide prospective longitudinal research infrastructure with standardised follow-up assessments, harmonized multimodal data collection (clinical, imaging, biosamples, and PROMs), and a modular platform architecture that supports future clinical, translational, and health services research projects across the entire stroke care continuum.The primary objective of Stroke-CORE is to establish a nationally harmonised, prospective cohort that provides comprehensive clinical data, imaging data, and biosamples. By combining longitudinal follow-up and multimodal data collection across all age groups and stroke subtypes, Stroke-CORE aims to address important evidence gaps in areas where effective prevention, treatment, and long-term management strategies remain limited, including intracerebral haemorrhage, post-stroke cognitive impairment and depression, secondary prevention, and paediatric stroke. The modular structure of Stroke-CORE and its topic-specific working groups of leading stroke experts in Germany within SN Stroke allow emerging research priorities and underrepresented areas of stroke research, to be systematically identified and translated into dedicated modules and future extensions of Stroke-CORE. By generating up-to-date and representative real-world data and providing a shared research infrastructure with minimal additional resource requirements, the cohort will facilitate translational, clinical, and health service research on stroke mechanisms, biomarkers, aetiology, prognosis, treatment pathways, and outcomes. In addition, Stroke-CORE will support the planning and feasibility assessment of future clinical trials by identifying suitable patient populations and generating hypotheses or providing the basis for sample size estimations. Hereby we report the protocol of Stroke-CORE.

## Methods

### Study design and setting

Stroke-CORE is constructed using a modular umbrella protocol. This architecture follows the principles of an adaptive research platform, allowing new modules, data streams, or biobanking initiatives to be incorporated over time without requiring revision of the core protocol.

**Fig. 1:**
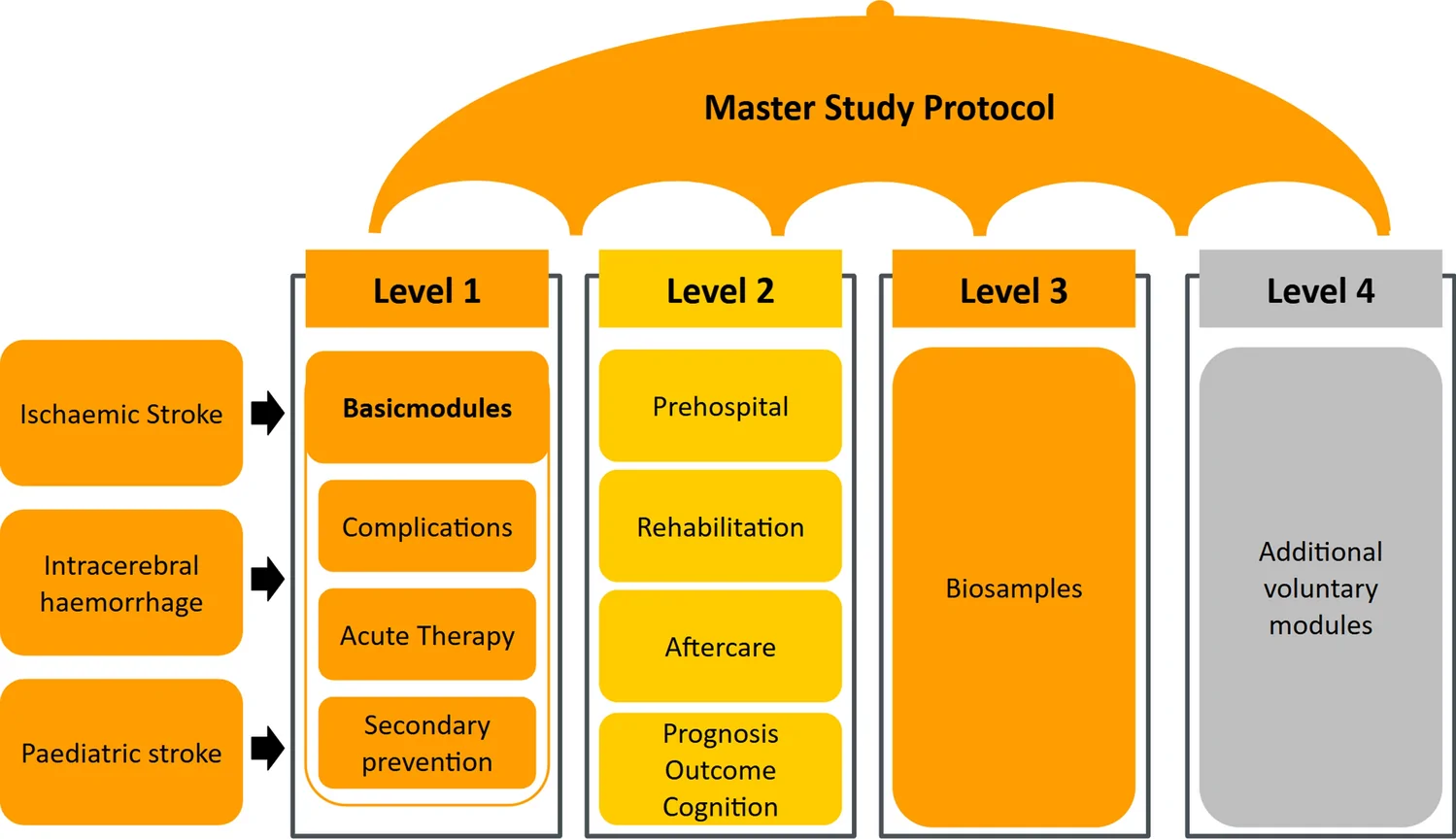
Modular architecture of SN Stroke

The cohort is structured in four levels. Level 1 comprises basic modules with a core data set collected at baseline, 90 days, and annually. Level 2 comprises expanded, pathway-specific modules with additional follow-up at discharge, 90 days, 180 days (optional), and annually. Level 3 is an additive module enabling collection of biosamples as part of routine treatment; and Level 4 comprises voluntary, separately funded and consented extensions defined and conducted by individual sites. All patients will be enrolled in one of the three Level 1 basic modules (ischaemic stroke, intracerebral haemorrhage, or paediatric stroke) and at least one of the Level 2 modules depending on the site (see Fig. 1).

The study operates within the established infrastructures of the Network University Medicine (NUM), including NUKLEUS (NUM Clinical Epidemiology and Study Platform), the NUM Methods- and Biosamples Hub (NUM-MB), the NUM Data Integration Centres (NUM-DICs), and the NUM Radiology Research Network (RACOON). Enrolment began in August 2026 following ethical approval, and is planned to continue until 30 June 2030, with interim analyses scheduled for mid-2027 and mid-2029.

Stroke-CORE is initially being implemented at twelve major German university hospitals with specialised stroke units. Of these, seven sites recruit adult stroke patients only, three sites enrol both paediatric and adult stroke patients, and two sites are dedicated exclusively to the recruitment of paediatric stroke patients. In subsequent implementation phases, the network is intended to expand to all German university hospitals and will also be open for enrolment at non-university hospitals.

### Participants

Eligible patients are identified and screened locally at each participating site by trained study personnel. The study targets a broad and representative population of stroke patients treated at participating centres, spanning the full clinical spectrum of ischaemic stroke and intracerebral haemorrhage in adults, and stroke in children.

**Fig. 2:**
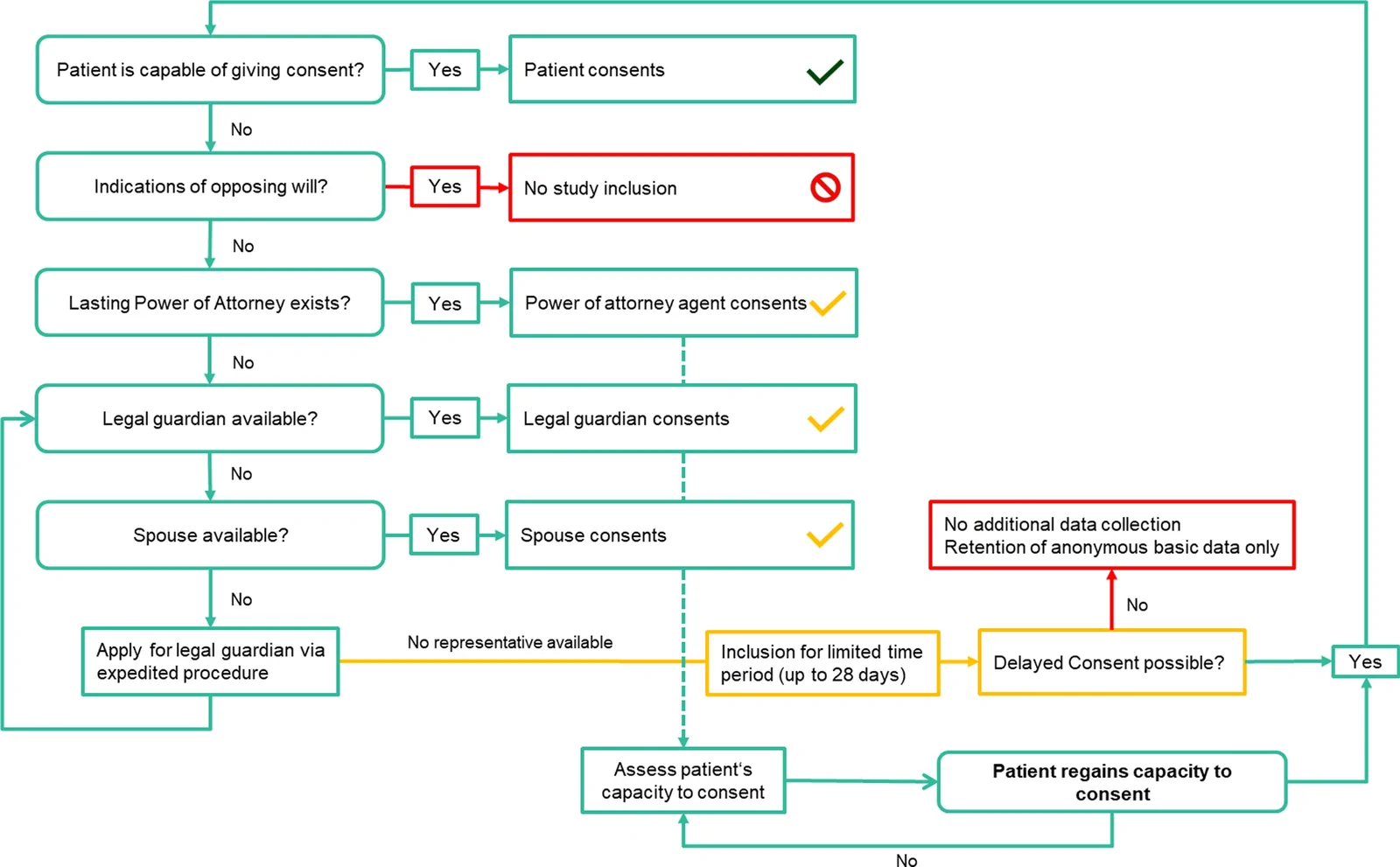
Consent process

The informed consent process is based on the Medical Informatics Initiative (MII) Broad Consent and an additional study-specific consent form [22–24]. To be able to also include patients who are unable to consent to study participation at the time, a process to obtain consent from an appropriate representative has been established (see Fig. 2). Subsequent reconsent will be sought from the patient whenever capacity is regained.

### Eligibility criteria

Patients are eligible if they meet all general inclusion criteria and none of the general exclusion criteria of the Master Study Protocol and additionally satisfy the criteria of at least one module. Additional, subtype- or domain-specific eligibility criteria are defined within each Module-specific annex Table 1.

**Table 1:** General eligibility criteria for the stroke-CORE Basic cohort

Type	Criterion	Description
Inclusion criteria	Diagnosis	Clinical or radiological diagnosis of acute ischaemic stroke or intracerebral haemorrhage: • Ischaemic stroke: focal neurological impairment of sudden onset, persisting more than 24 h, of presumed vascular origin, or radiological evidence of acute cerebral ischaemia corresponding to clinical symptoms • Intracerebral haemorrhage: clinical symptoms and radiological confirmation required
	Time window	Hospital admission within 24 h of symptom onset
	Age	≥18 years (adult basic Modules) or <18 years (paediatric basic Module)
	Consent	Signed informed consent from the patient or an appropriate representative
Exclusion criteria	Onset setting	In-hospital stroke onset
	Diagnosis	Transient ischaemic attack (TIA); subarachnoid haemorrhage (SAH)
	Aetiology	Stroke occurring as a complication of a medical procedure
	Concurrent participation	Concurrent enrolment in a randomised placebo-controlled interventional trial
	Prior participation	Prior enrolment in the Stroke-CORE basic cohort

### Recruitment

Each participating site contributes to the basic Level 1 module corresponding to the stroke subtypes it treats and selects at least one additional Level 2 module to participate in as soon as they are established. To support recruitment monitoring and quality assurance, each site maintains an anonymous, aggregated pre-screening log capturing all potentially eligible patients. The Stroke-CORE coordination provides sites with regular, automated feedback on recruitment performance benchmarked against anonymised data from other sites, supporting real-time identification of recruitment barriers.

## Data collection and data management

### Core data set

A basic set of clinical patient data, including socio-demographic data, epidemiological data, information on comorbidities, medical diagnoses, diagnostic procedures, therapies, results of imaging data, and patient-reported outcome measures will be documented. Data will be collected via an eCRF at all study sites based on a jointly agreed data collection protocol and stored in a central database. For Level 1, data collection is based on the quality assurance recommendations of the German Stroke Registers Study Group (Arbeitsgemeinschaft Deutschsprachiger Schlaganfall Register, ADSR) and the recommendation of the International Consortium for Health Outcomes Measurement (ICHOM) Stroke data set [25, 26]. Assessments are performed at baseline, discharge (or day 7 after admission, whichever occurs first), and 90 days, optionally 180 days follow-up after admission (Table 2). Long-term follow-up is conducted annually to ascertain survival and major health outcomes. Follow-up data include mortality, recurrent stroke and other vascular events, rehospitalisations, living situation, functional status, activities of daily living, and health-related quality of life. Outcome assessment is based on standardised instruments used in routine treatment, including the modified Rankin Scale (mRS) for functional outcome and ICHOM-recommended patient-reported outcome measures to facilitate national and international comparability of results.

**Table 2:** Follow-up schedule of the SN Stroke cohort and Levels. Numbers indicate the Level in which the respective actions are performed

Follow-Up	Baseline	Discharge	90-day Follow-up	180-day follow-up	Annual Follow-Up
Time point	Admission until discharge	Discharge/Admission +7 days (whichever comes first)	90 days post admission	180 days post admission	Annually post admission
Review of inclusion and exclusion criteria	1,2,3,4				
Informed consent process (IC)	1,2,3,4				
Registration of patients with the TTP	1,2,3,4				
**Clinical data**					
Anamnesis & documentation (signs & symptoms, clinical course, medical history, lab data, vital signs, treatment, outcome, etc. as detailed in eCRF incl. prehospital data)	1,2,3,4	1,2,3,4			
Comorbidities	1,2,4	1,2,4	4	2,4	4
Survival status	1,2,4	1,2,4	1,2,4	2,4	1,2,4
**Patient-reported outcomes**	1,2,4	1,2,4	1,2,4		4
Functional status		1,2,4	1,2,4	2,4	
Health related quality of life		1,2,4	1,2,4	2,4	
Living situation	1,2,4	1,2,4	1,2,4	2,4	
Lifestyle factors	1,2,4	1,2,4			
**Biosamples**					
Collection of residual biosamples from clinical routine diagnostics	3	3			
Collection of additional biosamples (prospective non-invasive samples) (**)	3	3	3	3	
Imaging data	1,2,3,4	1,2,3,4	4	4	

### Outcomes

Stroke-CORE assesses core outcomes on the entire stroke care pathway each assessed at defined time points from baseline through follow-up (Table 3). Comorbidities and vascular risk factors are documented according to predefined operational definitions based on the standardised variables of the ADSR as well as ICHOM Stroke. To minimise inter-site variability, detailed data dictionaries, standard operating procedures, harmonised eCRFs, and investigator training are provided to all participating centres. In addition, validation procedures and central plausibility checks are used to ensure consistent documentation across sites.

**Table 3:** Outcome measures

Outcomes	Exemplary variables	Scales and measurement tools
Disease severity	cognition, neurological deficits	NIHSS
Standard-of-care processes	Imaging, dysphagia screening, ECG monitoring, thrombolysis, thrombectomy, rehabilitation	time-to-treatment measures, and process indicators based on ADSR-questionnaire/guideline adherence
In-hospital complications	pneumonia, haemorrhage, oedema, sepsis, delirium, epilepsy	
Neuropsychiatric sequelae	depression/anxiety, cognitive impairment	PHQ-4 MoCA
Recurrent ischaemic or haemorrhagic stroke	Recurrent stroke	
Comorbidities	Comorbidities, (cardiovascular) risk factors, medication burden, feeding tube, ventilation, medication	Comorbidity indices
Health-Related Quality of Life	Mobility, self-care, usual activities, pain, anxiety/depression, social participation, perceived health, fatigue, pain intensity, emotional well-being	EQ5D-3L PROMIS-10 Global Health
Functional status	Ambulation (pre/post stroke), toileting (pre/post stroke), dressing (pre/post stroke), communication, living location, living alone, discharge destination	mRS
Survival status	Vital status, date of death	

### Clinical data management

Clinical patient data are documented and stored in the secure secuTrial® database provided by the NUKLEUS Clinical Data Management Unit. Study personnel access and manage site-specific data through role-based permissions administered by the Trusted Third Party (TTP). Server infrastructure is maintained with regular security updates, firewall protection, threat detection, and daily on-site and off-site backups. Clinical and imaging data are linked using project-specific pseudonyms managed by the TTP, while personally identifiable information remains exclusively at the recruiting sites. The reuse of data for further research projects is governed by the central Use and Access Committee (UAC) of the NUM.

### Neuroradiological data

Imaging data from routine clinical examinations of each participant (including CT/CT-angiography and, where available, MRI, angiography, or DSA) will be collected and uploaded to RACOON CENTRAL, a cloud from the Radiological Research Network (RACOON), using only pseudonymised identifiers and are hosted according to the NUM standards. Following acquisition at the local sites, imaging data are first processed on the decentralised RACOON-NODES, where identifying metadata are removed and MRI data are defaced prior to transfer to RACOON-CENTRAL to further protect participant privacy and minimise the risk of facial reconstruction and re-identification. Subsequently, the data are transferred to RACOON-CENTRAL in pseudonymised form. Imaging data are stored in the international DICOM (Digital Imaging and Communications in Medicine) standard and are accessible via DICOM interfaces. Structured reporting data and clinical study data are maintained in Mint Central and linked to the imaging data.

### Biosamples

Standards to ensure high and consistent quality of biosamples across all sites will be developed in collaboration with the Biosample Core Unit of NUM-MB (BCU) and detailed in the Biobanking SOP of the SN Stroke. The BCU conducts training sessions at the study sites to communicate the content of the finalised, mandatory SOP. In addition, the biobank audits conducted by the BCU ensure compliance with the SOP at all participating sites [9, 27].

### Quality assurance

Data quality is ensured through a combination of automated validation procedures and central oversight mechanisms. Data entered into the electronic case report form (eCRF) are automatically checked for completeness, consistency, and plausibility. In addition, the Stroke-CORE Coordination continuously monitors recruitment and data quality through automated data pipelines. Participating sites receive regular feedback on recruitment performance, benchmarked against anonymised data from other centres. Study-specific quality and performance indicators, derived automatically from clinical documentation and biosample data, are used to assess documentation quality, biosample completeness and quality, and overall recruitment performance. These indicators support continuous quality improvement and may, in the future, facilitate the identification of sites suitable for participation in embedded studies or clinical trials. Audits and core analysis will predominantly be conducted by the Epidemiological Core Unit (ECU) of the NUM-MB.

### Statistical methods

While most cohort analyses will be defined as part of later use cases, this protocol pre-defines certain uses that will not require future approval by the Use and Access Committee. These include regular descriptive analyses of the cohort, for example for scientific presentations and cohort description publications, analysis of data completeness and site participation, to evaluate data completeness and potential selection bias, and regular analysis and visualisation of meta-data and structure, process, and outcome indicators reflecting the performance of participating infrastructures, sites, and external partners. In addition, the specific research questions defined in the modules and the ongoing monitoring of the type of consents obtained also do not require approval.

### Sample size calculation

Sample size considerations are guided by the aim of developing predictive models of varying complexity within specific stroke subgroups for a range of outcomes. A total of approximately 12,750 patients (including anticipated dropouts) are expected to be enrolled in the study over the planned enrolment time of 50 months across ten to twelve participating sites, corresponding to an average recruitment rate of about 255 patients per month, or approximately 25 patients per site per month. Assuming a dropout rate of up to 20% [28–31], a final analysis population of approximately 10,200 patients is expected.

This sample size is considered sufficient to support development of prediction models even for comparatively rare stroke subtypes (e.g., prevalence of 10%) and for outcomes with a low event rate (e.g., 10% at three months), consistent with current recommendations requiring at least 100 events for a robust prediction model. The projected sample additionally provides adequate power (80% at a two-sided significance level of 5%) to detect differences in outcome of 5–10 percentage points between subgroups occurring with a prevalence as low as 5%. The feasibility of these recruitment targets is supported by the scale of the participating network: the university hospitals and NUM partners involved collectively treat more than 50,000 stroke patients annually in specialised stroke units.

### Trial registration

The protocol is registered at German Clinical Trials Register (DRKS00039775, July 8th, 2026).

## Perspective

Stroke-CORE was established to address a long-standing lack of harmonised nationwide infrastructures for stroke research in Germany. Its modular umbrella design provides long-term flexibility, allowing disease- and domain-specific modules to be added, modified, or discontinued without requiring amendments to the overarching master protocol. This structure enables the cohort to adapt efficiently to emerging scientific questions and evolving research priorities.

A key feature of Stroke-CORE is its commitment to representative patient inclusion. Patients with severe stroke are frequently underrepresented in prospective studies because acute neurological deficits often preclude informed consent at the time of enrolment. To address this challenge, Stroke-CORE implements a structured consent framework that includes proxy consent procedures where appropriate, enabling the inclusion of patients who are temporarily unable to provide consent themselves. Subsequent consent from the patient is obtained whenever possible. Systematic monitoring of consent procedures further supports the recruitment of a cohort that reflects the full spectrum of stroke severity encountered in routine clinical care.

Beyond its research function, the pre-screening infrastructure offers opportunities for near real-time monitoring of stroke-related hospitalisations across participating university hospitals. This may facilitate the early identification of changes in stroke incidence, patient characteristics, and treatment patterns, thereby generating insights relevant to healthcare planning and service delivery.

Lastly, Stroke-CORE is intended to function as a durable national research resource. The platform provides a shared, quality-assured foundation of clinical, imaging, and biosample data that can support future clinical trials, biomarker studies, translational research, health services research, and investigator-initiated extensions. Its Dynamic Use & Access governance framework is designed to promote broad scientific use.

A potential limitation of the initial implementation phase is that recruitment is currently restricted to university hospitals. Although the participating centres provide comprehensive stroke unit care to the region of broadly comparable size, recruitment volumes and patient characteristics may vary between sites. Therefore, recruitment performance and key patient characteristics will be monitored centrally throughout the study. Furthermore, representativeness and potential selection bias will be assessed through comparisons with ADSR registry data. The initial implementation at university hospitals may result in an overrepresentation of patients with more severe stroke sequelae. To improve representativeness, Stroke-CORE is planned to expand data collection also to non-university hospitals for which reimbursement via case payment is planned.

### Funding sustainability

Long-term sustainability of large-scale observational cohorts requires stable financial support beyond the initial funding period. Stroke-CORE currently benefits from secured funding until 2030 and is embedded within the Network University Medicine (NUM), which provides a sustainable national research infrastructure. In addition, the cohort is designed as a shared research platform for investigator-initiated and externally funded projects.

### Site expansion

During the initial funding phase, Stroke-CORE will be established at 12 university stroke centres across Germany. The network is designed to expand in a stepwise manner to include all German university hospitals over time as well as to expand to further non-university hospitals.

## Conclusion

Stroke-CORE establishes a national, prospective, longitudinal, multi-centre cohort with a decentralised biobank, encompassing adult ischaemic stroke, adult intracerebral haemorrhage, as well as paediatric stroke across the entire continuum of care. Through its modular umbrella design, levelled data and biosample collection, and harmonised NUM-based infrastructure, the cohort aims to provide a durable, adaptive platform for translational, outcomes, and health-services research in stroke. By enabling the inclusion of patients with severe acute stroke through a dedicated consent framework, Stroke-CORE aims to facilitate the recruitment of a cohort that is representative of real-world stroke populations. As a sustainable national stroke research platform, it is expected to strengthen collaborative stroke research in Germany, support the design and implementation of future clinical trials, and accelerate the generation of evidence to improve stroke prevention, treatment, and long-term care.

## Data Availability

No datasets were generated or analysed during the current study.

## References

[1] Wiegand F. Häufigkeit des Schlaganfalles in Deutschland 2023: Schätzungen der Absolutzahlen und Mortalität für die Versorgungsplanung. DSG-Kongress 2025; Berlin 2025.

[2] Busch, M., & Kuhnert, R. (2017). 12-Month prevalence of stroke or chronic consequences of stroke in Germany. *J Health Monit.,**2*, 64–69. 10.17886/RKI-GBE-2017-019 PMC10161268 37151304

[3] Brüggenjürgen, B., Rupprecht, H.-J., Willich, S., Spannagl, M., Ehlken, B., Smala, A., Berger, K., & Diener, H. (2005). Cost of atherothrombotic diseases—myocardial infarction, ischaemic stroke and peripheral arterial occlusive disease—in Germany. *Journal of Public Health,**13*, 216–224. 10.1007/s10389-005-0112-3

[4] Kolominsky-Rabas, P. L., Heuschmann, P. U., Marschall, D., Emmert, M., Baltzer, N., Neundörfer, B., Schöffski, O., & Krobot, K. J. (2006). Lifetime cost of ischemic stroke in Germany: Results and national projections from a population-based stroke registry: The Erlangen Stroke Project. *Stroke,**37*(5), 1179–1183. 10.1161/01.STR.0000217450.21310.90 16574918

[5] Rossnagel, K., Nolte, C. H., Muller-Nordhorn, J., Jungehulsing, G. J., Selim, D., Bruggenjurgen, B., Villringer, A., & Willich, S. N. (2005). Medical resource use and costs of health care after acute stroke in Germany. *European Journal of Neurology,**12*(11), 862–868. 10.1111/j.1468-1331.2005.01091.x 16241975

[6] Dodel, R. C., Haacke, C., Zamzow, K., Paweilik, S., Spottke, A., Rethfeldt, M., Siebert, U., Oertel, W. H., Schöffski, O., & Back, T. (2004). Resource utilization and costs of stroke unit care in Germany. *Value Health.,**7*(2), 144–152. 10.1111/j.1524-4733.2004.72314.x 15164804

[7] Ridwan, S., Urbach, H., Greschus, S., von Hagen, J., Esche, J., & Boström, A. (2017). Health care costs of spontaneous aneurysmal subarachnoid hemorrhage for rehabilitation, home care, and in-hospital treatment for the first year. *World Neurosurgery,**97*, 495–500. 10.1016/j.wneu.2016.09.123 27744076

[8] Gesundheitsberichterstattung des Bundes (GBE). Schlaganfall Krankheitskosten je Einwohner in Euro in Deutschland Berichtsjahr 2023 2023. Available from: https://www.gbe-bund.de/gbe/isgbe.news?p_sprache=D&p_news_id=1406&p_typ=N. Accessed: 07.10.2026.

[9] Goyal, M., Menon, B., van Zwam, W., Dippel, D., Mitchell, P., Demchuk, A., Dávalos, A., Majoie, C., van der Lugt, A., de Miquel, M., Donnan, G., Roos, Y., Bonafe, A., Jahan, R., Diener, H., van den Berg, L., Levy, E., Berkhemer, O., Pereira, V., … Jovin, T. (2016). Endovascular thrombectomy after large-vessel ischaemic stroke: A meta-analysis of individual patient data from five randomised trials. *Lancet,**387*(10029), 1723–1731. 10.1016/S0140-6736(16)00163-X 26898852

[10] GBD 2019 Stroke Collaborators. (2021). Global, regional, and national burden of stroke and its risk factors, 1990-2019: A systematic analysis for the Global Burden of Disease Study 2019. *Lancet Neurology,**20*(10), 795–820. 10.1016/S1474-4422(21)00252-0 PMC8443449 34487721

[11] Dunbar, M., & Kirton, A. (2018). Perinatal stroke: Mechanisms, management, and outcomes of early cerebrovascular brain injury. *Lancet Child & Adolescent Health,**2*(9), 666–676. 10.1016/S2352-4642(18)30173-1 30119760

[12] Ferriero, D., Fullerton, H., Timothy, J., Bernard, T., Billinghurst, L., Daniels, S., DeBaun, M., deVeber, G., Ichord, R., Jordan, L., Massicotte, P., Meldau, J., Roach, E., & Smith, E. (2019). Management of stroke in neonates and children: a scientific statement from the American Heart Association/American Stroke Association. *Stroke*. 10.1161/STR.0000000000000183 30686119

[13] Ahmadi, M., Laumeier, I., Ihl, T., Steinicke, M., Ferse, C., Endres, M., Grau, A., Hastrup, S., Poppert, H., Palm, F., Schoene, M., Seifert, C., Kandil, F., Weber, J., von Weitzel-Mudersbach, P., Wimmer, M., Algra, A., Amarenco, P., Greving, J., … Audebert, H. (2020). A support programme for secondary prevention in patients with transient ischaemic attack and minor stroke (INSPiRE-TMS): An open-label, randomised controlled trial. *Lancet Neurology,**19*(1), 49–60. 10.1016/S1474-4422(19)30369-2 31708447

[14] Bendszus, M., Fiehler, J., Subtil, F., Bonekamp, S., Aamodt, A., Fuentes, B., Gizewski, E., Hill, M., Krajina, A., Pierot, L., Simonsen, C., Zeleňák, K., Blauenfeldt, R., Cheng, B., Denis, A., Deutschmann, H., Dorn, F., Flottmann, F., Gellißen, S., … Thomalla, G. (2023). Endovascular thrombectomy for acute ischaemic stroke with established large infarct: Multicentre, open-label, randomised trial. *Lancet,**402*(10414), 1753–1763. 10.1016/S0140-6736(23)02032-9 37837989

[15] Ebinger, M., Siegerink, B., Kunz, A., Wendt, M., Weber, J., Schwabauer, E., Geisler, F., Freitag, E., Lange, J., Behrens, J., Erdur, H., Ganeshan, R., Liman, T., Scheitz, J., Schlemm, L., Harmel, P., Zieschang, K., Lorenz-Meyer, I., Napierkowski, I., … Audebert, H. (2021). Association between dispatch of mobile stroke units and functional outcomes among patients with acute ischemic stroke in Berlin. *JAMA,**325*(5), 454–466. 10.1001/jama.2020.26345 PMC7856548 33528537

[16] Schwarzbach, C., Eichner, F., Rücker, V., Hofmann, A., Keller, M., Audebert, H., von Bandemer, S., Engelter, S., Geis, D., Gröschel, K., Haeusler, K., Hamann, G., Meisel, A., Sander, D., Schutzmeier, M., Veltkamp, R., Heuschmann, P., & Grau, A. (2023). The structured ambulatory post-stroke care program for outpatient aftercare in patients with ischaemic stroke in Germany (SANO): An open-label, cluster-randomised controlled trial. *Lancet Neurology,**22*(9), 787–799. 10.1016/S1474-4422(23)00216-8 37459876

[17] Thomalla, G., Simonsen, C., Boutitie, F., Andersen, G., Berthezene, Y., Cheng, B., Cheripelli, B., Cho, T., Fazekas, F., Fiehler, J., Ford, I., Galinovic, I., Gellissen, S., Golsari, A., Gregori, J., Günther, M., Guibernau, J., Häusler, K., Hennerici, M., … Gerloff, C. (2018). MRI-guided thrombolysis for stroke with unknown time of onset. *New England Journal of Medicine,**379*(7), 611–622. 10.1056/NEJMoa1804355 29766770

[18] Broderick, J. P., Palesch, Y. Y., & Janis, L. S. (2016). The National Institutes of Health StrokeNet: A user’s guide. *Stroke,**47*(2), 301–303. 10.1161/strokeaha.115.011743 PMC4729626 26715457

[19] StrokeLink. National Network of Clinical Research on Stroke 2026. Available from: https://strokelink-avc.fr/en. Accessed: 25.08.2026.

[20] NIHR (National Institute for Health and Care Research). Research Delivery Network 2026. Available from: https://www.nihr.ac.uk/support-and-services/support-for-delivering-research/research-delivery-network. Accessed: 25.08.2026.

[21] UK Clinical Research Collaboration (UKCRC). Clinical Research Networks 2026. Available from: https://www.ukcrc.org/research-infrastructure/clinical-research-networks/. Accessed: 25.08.2026.

[22] Medical Informatics Initiative Germany (Medizininformatik-Initiative). Template text for patient consent forms 2026. Available from: https://www.medizininformatik-initiative.de/en/template-text-patient-consent-forms. Accessed: 25.08.2026.

[23] Zenker, S., Strech, D., Ihrig, K., Jahns, R., Müller, G., Schickhardt, C., Schmidt, G., Speer, R., Winkler, E., von Kielmansegg, S. G., & Drepper, J. (2022). Data protection-compliant broad consent for secondary use of health care data and human biosamples for (bio)medical research: Towards a new German national standard. *Journal of Biomedical Informatics,**131*, Article 104096. 10.1016/j.jbi.2022.104096 35643273

[24] Zenker, S., Strech, D., Jahns, R., Müller, G., Prasser, F., Schickhardt, C., Schmidt, G., Semler, S. C., Winkler, E., & Drepper, J. (2024). National standardisierter Broad Consent in der Praxis: Erste Erfahrungen, aktuelle Entwicklungen und kritische Betrachtungen. *Bundesgesundheitsblatt - Gesundheitsforschung - Gesundheitsschutz,**67*(6), 637–647. 10.1007/s00103-024-03878-6 PMC11166792 38639817

[25] Hillmann, S., Wiedmann, S., Rücker, V., Berger, K., Nabavi, D., Bruder, I., Koennecke, H. C., Seidel, G., Misselwitz, B., Janssen, A., Burmeister, C., Matthis, C., Busse, O., Hermanek, P., & Heuschmann, P. U. (2017). Stroke unit care in Germany: The German stroke registers study group (ADSR). *BMC Neurology,**17*(1), Article 49. 10.1186/s12883-017-0819-0 PMC5343401 28279162

[26] Rimmele, D. L., Lebherz, L., Frese, M., Appelbohm, H., Bartz, H. J., Kriston, L., Gerloff, C., Härter, M., & Thomalla, G. (2019). Outcome evaluation by patient reported outcome measures in stroke clinical practice (EPOS) protocol for a prospective observation and implementation study. *Neurology Research and Practice,**1*, Article 28. 10.1186/s42466-019-0034-0 PMC7650080 33324894

[27] Mishra, A., Malik, R., Hachiya, T., Jürgenson, T., Namba, S., Posner, D., Kamanu, F., Koido, M., Le Grand, Q., Shi, M., He, Y., Georgakis, M., Caro, I., Krebs, K., Liaw, Y., Vaura, F., Lin, K., Winsvold, B., Srinivasasainagendra, V., … Debette, S. (2022). Stroke genetics informs drug discovery and risk prediction across ancestries. *Nature,**611*(7934), 115–123. 10.1038/s41586-022-05165-3 PMC9524349 36180795

[28] ClinicalTrials.gov. Validate: Trustworthy AI to Improve Acute Stroke Outcomes (VALIDATE) ClinicalTrials.gov ID: NCT05622539 2022. Available from: https://clinicaltrials.gov/study/NCT05622539. Accessed: 08.10.2026.

[29] Cordes, D., Gerloff, C., Heise, K.-F., Hummel, F. C., Schulz, R., Wolf, S., Haevernick, K., Krüger, H., Krause, L., Suling, A., Wegscheider, K., Zapf, A., Dressnandt, J., Schäpers, B., Schrödl, C., Hauptmann, B., Kirchner, A., Brault, A., Gutschalk, A., … Voller, B. (2024). Efficacy and safety of transcranial direct current stimulation to the ipsilesional motor cortex in subacute stroke (NETS): A multicenter, randomized, double-blind, placebo-controlled trial. *The Lancet Regional Health - Europe,**38*, Article 100825. 10.1016/j.lanepe.2023.100825 PMC10928272 38476746

[30] Heuschmann, P. U., Montellano, F. A., Ungethüm, K., Rücker, V., Wiedmann, S., Mackenrodt, D., Quilitzsch, A., Ludwig, T., Kraft, P., Albert, J., Morbach, C., Frantz, S., Störk, S., Haeusler, K. G., & Kleinschnitz, C. (2021). Prevalence and determinants of systolic and diastolic cardiac dysfunction and heart failure in acute ischemic stroke patients: The SICFAIL study. *ESC Heart Failure,**8*(2), 1117–1129. 10.1002/ehf2.13145 PMC8006617 33350167

[31] Wahab, A., Nadarajah, R., Reynolds, C., Bennett, S., Ambakederemo, E., Harris, M., Younsi, T., Joseph, T., Raveendra, K., Smith, A., Bhatty, A., Lip, G. Y. H., Swoboda, P. P., Wu, J., & Gale, C. P. (2024). Phenotypic characterization of people at risk of atrial fibrillation: Protocol for the FIND-AF longitudinal cohort study. *European Journal of Preventive Cardiology,**31*(18), 2099–2103. 10.1093/eurjpc/zwae303 PMC11663481 39319414

